# Microbiota regulation of viral infections through interferon signaling

**DOI:** 10.1016/j.tim.2022.01.007

**Published:** 2022-02-06

**Authors:** Nurul I. Wirusanti, Megan T. Baldridge, Vanessa C. Harris

**Affiliations:** 1 Department of Global Health – Amsterdam Institute for Global Health and Development (AIGHD), Amsterdam University Medical Center, Academic Medical Center, Amsterdam, The Netherlands; 2 Division of Infectious Diseases and Center for Experimental and Molecular Medicine (CEMM), Department of Medicine, Amsterdam University Medical Center, Academic Medical Center, Amsterdam, The Netherlands; 3 Division of Infectious Diseases, Department of Medicine, Edison Family Center for Genome Sciences & Systems Biology, Washington University School of Medicine, St Louis, MO, USA; 4 Department of Molecular Microbiology, Washington University School of Medicine, St Louis, MO, USA; 5 All the authors wrote and edited the manuscript

## Abstract

The interferon (IFN) response is the major early innate immune response against invading viral pathogens and is even capable of mediating sterilizing antiviral immunity without the support of the adaptive immune system. Cumulative evidence suggests that the gut microbiota can modulate IFN responses, indirectly determining virological outcomes. This review outlines our current knowledge of the interactions between the gut microbiota and IFN responses and dissects the different mechanisms by which the gut microbiota may alter IFN expression to diverse viral infections. This knowledge offers a basis for translating experimental evidence from animal studies into the human context and identifies avenues for leveraging the gut microbiota–IFN–virus axis to improve control of viral infections and performance of viral vaccines.

## The triangular relationship between the host, the gut microbiota, and viral pathogens

Coevolution of the **intestinal microbiota** (see Glossary) and the host innate immune system has resulted in a delicate balance of reciprocal interactions to maintain homeostasis in the gut. The microbiota plays a fundamental role in the induction, training, and maintenance of the host immune system [1]. At the same time, the immune system has evolved to constrain and maintain a symbiotic relationship with these microbes [2]. A critical role for the gut microbiota in shaping host immunity becomes readily apparent in the context of eukaryotic viral infections. On the one hand, the gut microbiota can directly interact with virus particles and influence their infectivity. Numerous studies have described the mechanisms by which the gut microbiota is able to promote viral replication and pathogenesis (reviewed in [3,4]). On the other hand, the gut microbiota can prime and activate host antiviral immunity [5]. Together, the gut microbiota, host immunity, and viral pathogens interact in a complex triangular relationship to determine infection and disease outcomes.

One recurring mechanism by which the gut microbiota has been found to influence antiviral control is via modulation of the IFN response. IFNs are a class of cytokines, secreted by host cells upon viral infection, that have a potent antiviral activity [6,7]. IFNs have dual actions: first, they induce an immediate antiviral state in infected and neighboring cells, and second, they link the innate and adaptive immunity, mainly through priming of dendritic cells (DCs) [7,8]. Evidence to date indicates that the gut microbiota can either promote or suppress IFN signaling, depending on the specific virus and setting [9–19]. Interestingly, the influence of the gut microbiota on IFN responses appears to be conserved across a wide range of viruses and, in parallel, numerous bacteria in the microbiota and their byproducts can activate IFN signaling. Understanding how the microbiota controls IFN responses will be critical to inform novel antiviral and viral vaccination strategies.

This review provides a brief overview of IFN biology followed by a detailed delineation of how the gut microbiota has been shown to modulate antiviral IFN responses at both local and remote sites, discussing the specific mechanisms underlying microbiota and IFN interactions during viral infections. We focus on the gut microbiota, although these microbiota-driven mechanisms are likely at play across diverse anatomic sites, such as the lung and skin. Finally, the implications of these host–microbiota–viral pathogen interactions for antiviral therapies and viral vaccination strategies are explored.

## Brief overview of IFN responses

The IFN cytokine family contains three distinct types of IFN – types I, II, and III – with antiviral activity associated mainly with type I IFN and type III IFN, which we focus upon here [20]. The type I IFN family consists of IFN-α, IFN-β, IFN-ε, IFN-κ, IFN-δ, IFN-ω, IFN-ζ (mice), and IFN-τ (ruminants), though IFN-α and IFN-β are the best studied [21]. These cytokines are broadly associated with protection against systemic viral infections and contribute to restriction of infection at mucosal sites [7]. Type III IFNs, consisting of four different subtypes of IFN-λ, play a more prominent role in the protection of mucosal sites such as the intestinal and respiratory tracts [22].

Upon viral infection, the expression of IFNs is triggered by the sensing of viral nucleic acid by a variety of **pattern-recognition receptors** (**PRRs**). Both extracellular receptors, such as the membrane-bound Toll-like receptors (TLRs), and cytosolic receptors, such as RIG-I-like receptors (RLRs) and DNA sensors [which include cyclic GAMP synthase (cGAS)], can activate the expression of IFNs [8]. Subsequently, released IFNs bind to their respective receptors in an autocrine manner (the infected cell itself) or a paracrine manner (the neighboring cells) [23]. Type I IFNs bind to a heterodimer receptor complex composed of IFNAR1 and IFNAR2, while type III IFNs (or IFN-λ) bind to a heterodimer receptor complex comprised of IFNLR1 and IL-10RB. Despite distinct receptor complexes, type I and III IFNs share similar signaling cascades involving the phosphorylation of the JAK/STAT pathway and the translocation and binding of interferon regulatory factors (IRFs) to IFN-stimulated response elements (ISREs), inducing the expression of **IFN-stimulated genes** (**ISGs**) [24]. Expression of ISGs drives an antiviral state in infected and uninfected neighboring cells, resulting in direct interference with viral replication and dissemination [25].

Emerging paradigms suggest that type I and III IFNs each have unique and distinct roles in controlling viral infection. IFN-λ plays a more prominent role in the protection of mucosal sites, whereas IFN-α/β is more involved in the control of systemic infections. The localized function of IFN-λ results from the limited expression of IFNLR1 subunit of the type III IFN receptor to predominantly epithelial cells and only a subset of immune cells. By contrast, the receptor subunits for type I IFN, IFNAR1 and IFNAR2, are broadly expressed in nearly all nucleated cells [26]. Moreover, the IFN-α/β response is characterized by a rapid increase but also rapid decline of high-magnitude ISG expression, while ISG expression induced by IFN-λ is of lower magnitude, more delayed, but more sustained [27]. The differential localization and kinetics of IFN-λ responses may confine antiviral responses to mucosal sites without inducing excessive inflammatory responses systemically, unless local responses fail to curb the infection [21]. Comprehensive comparisons between IFN-α/β and IFN-λ for specific viral infections are available elsewhere [8,24,28].

In addition to directly inducing an antiviral state, IFNs are also important regulators of the adaptive arm of the immune system. Nearly all immune cells express IFNAR1 and IFNAR2 and are therefore responsive to type I IFN. Conventional DCs (cDC) in particular rely on cues from type I IFNs for functional maturation and migration [29]. Functional cDCs are important in the priming of both T cells and B cells. Type I IFNs can also directly signal T cells to become activated and proliferate [30]. The formation of germinal-center B cells and subclass distribution of IgG also depends on IFN-α/β signaling [31]. The role of IFN-λ in regulating adaptive immunity is only beginning to be understood. The extent to which human immune cells express IFNLR1, and thereby respond to IFN-λ stimulation, remains controversial. Naïve B cells are responsive to IFN-λ and require IFN-λ signaling to differentiate into plasmablasts and become functionally active, permitting cytokine release and antibody production [32]. CD8^+^ T cells do not directly respond to IFN-λ but still require IFN-λ to modulate the activation, antigen uptake, and migration of lung DCs [33]. In addition, IFN-λ can indirectly regulate T cell and B cell responses through the thymic stromal lymphopoietin (TSLP) axis, a cytokine produced by **M cells** that is important for adaptive immune regulation [34]. In summary, IFNs are powerful antiviral cytokines, playing a central role in orchestrating innate and adaptive immune responses to viral infection.

## The gut microbiota modulates IFN responses locally and remotely

The gastrointestinal tract houses the body’s most densely populated microbiota, and thereby signals from the gut microbiota influence mucosal immune responses to enteric virus infections [35]. However, it is also widely accepted that the gut microbiota can influence sites remote from the gastrointestinal tract. Correspondingly, the gut microbiota modulation of IFN responses occurs not only locally but also at extraintestinal compartments (Box 1). The means by which the resident intestinal microbiota influences distal sites remains an active area of study. There are at least two possible mechanisms: (i) commensal bacterial products or metabolites enter the systemic circulation and reach distal sites where they prime residing immune and epithelial cells; and (ii) circulating immune cells sample components of the gut microbiota in the intestine then migrate to other parts of the body to influence the local immune response. Further defining the mechanisms of crosstalk between the gut microbiota and immune response will be essential to harnessing the potential of the gut microbiota in antiviral immunity.

## Potential mechanisms underlying gut microbiota–IFN–viral interactions

Prior studies have uncovered a variety of mechanisms by which the gut microbiota modulates IFN responses, delineating different components of the bacterial microbiota that contribute to these interactions. Here, we outline three key mechanistic themes underpinning microbiota–IFN interactions in relation to viral infections: microbiota-mediated control of homeostatic IFN; microbiota-derived PRR ligands that induce IFN activation; and microbiota-derived metabolites that regulate IFN expression (Figure 1, Key figure). It is important to note that these mechanistic themes do not work in isolation but rather they are interconnected and overlapping with each other. Homeostatic IFN production, for instance, may be maintained by bacterial ligands and/or metabolites. In turn, these ligands and metabolites can influence and alter microbiota composition as well [36].

### The gut microbiota controls homeostatic IFN and ISG expression

Current evidence points to a role of the microbiota in influencing and maintaining IFN responses before infections occur [10,18,19,37]. The gut microbiota modulates the expression of ‘homeostatic IFN expression’, a constitutive basal IFN expressed at a very low level that is crucial for timely activation of IFN antiviral activity upon infection [10,18,19,37]. Often, homeostatic ISG expression is used as a surrogate for IFN expression due to the extremely low and difficult-to-detect levels of IFNs in this basal state [37]. Germ-free (GF) or antibiotic-treated mice devoid of residing gut microbiota have altered basal type I and type III IFN expression and signaling, predisposing these mice to defective or delayed viral clearance following infection [10,18,19,37].

Plasmacytoid DCs (pDCs) are a subclass of DCs that are particularly important for the production of type I IFN. The bacterial microbiota can play a role in controlling the expression of homeostatic type I IFNs by pDCs, which is required for transcriptional, epigenetic, and metabolic programming of cDCs [10]. cDCs isolated from GF mice, when compared to cDCs isolated from control specific-pathogen-free (SPF) mice, lack numerous H3K4me3 **epigenetic markers** (indicative of transcriptionally active regions) for type I IFNs and ISGs. As a consequence, when cDCs from GF mice are stimulated with **poly I:C** (viral antigen) or **LPS** (bacterial antigen), transcription factors downstream of PRR activation can translocate to the nucleus but fail to bind to the promoter regions of these genes [18]. Impaired cDC programming and maturation therefore likely leads to an inability of cDCs to optimally prime CD8^+^ T cell and natural killer (NK) cell responses.

Similarly, macrophages also depend on instructive signals from the bacterial microbiota to maintain homeostatic IFN responses. In murine models of lymphocytic choriomeningitis virus (LCMV) or influenza infection, depletion of the gut microbiota results in macrophages that are unresponsive to virus or IFN-β stimulation [19]. Further, genome-wide transcriptional analysis of these macrophages reveals that IFN-related genes, including PRRs and ISGs, are downregulated. This impaired homeostatic IFN signaling translates to defects in induction of adaptive immune responses when mice are infected with either LCMV or influenza virus, such that CD8^+^ T cell function and antigen-specific antibody production are both impaired [19]. Similar observations have been made for encephalomyocarditis virus (EMCV) infection, wherein antibiotic-treated mice display unresponsive macrophages, along with impaired NK cell toxicity and decreased type I IFN and ISG expression, with reduced survival and exacerbated disease phenotypes [15]. Interestingly, signals from the gut microbiota of conventional mice are able to limit EMCV replication in distal target cells in the brain to protect them from neurological pathogenicity [15]. Altogether, these studies underscore the likely importance of gut microbiota-derived signals in influencing homeostatic type I IFN responses and viral control at extraintestinal sites.

At mucosal sites, nonhematopoietic epithelial and stromal cells contribute significantly to the maintenance of mucosal immunity alongside immune cells. Nonhematopoietic cells are also equipped with PRRs to sense pathogens and produce cytokines [38,39]. Lung stromal cells, for example, respond to tonic type I IFN during homeostasis and produce basal ISGs in a microbiota-dependent manner. The source of type I IFN in the lung remains an open question. Type I IFN signaling in the lung induces basal ISG expression in both stromal and hematopoietic cells; when the gut microbiota is absent, ISG expression is disrupted in lung stromal cells but not in immune cells for unclear reasons [11]. It is possible that the regulation of baseline IFN and ISGs in mucosal compartments and systemic compartments is distinct. However, given the heterogeneity in microbiota models (antibiotic-treated versus GF mice), it also possible that differences in experimental models have led to observed discrepancies.

There is currently limited knowledge of the role of the gut microbiota in homeostatic IFN-λ signaling. Only one study so far has evaluated IFN regulation of basal ISG expression in the intestinal tract of mice [37]; it found that, in an antibiotic-treatment model, microbiota-associated IFN-λ signaling drives expression of homeostatic ISGs in a nonuniform manner along the intestinal epithelium. Specifically, IFN-λ-driven tonic ISG expression is localized to the tips of the intestinal villi, suggesting preferential expression of homeostatic ISGs by mature enterocytes. The physiological relevance of this preferential localized expression of microbiota-induced homeostatic ISGs remains to be further investigated but does appear to be required to mount a timely and robust antiviral response against enteric viruses that target mature enterocytes, such as MRV [37]. To date, no study has assessed homeostatic IFN-λ expression in airway epithelia. Considering the significance of IFN-λ in antiviral defense against respiratory pathogens, and the capacity of the gut microbiota to induce type I IFN expression at extraintestinal sites, homeostatic IFN-λ expression in the airway in response to microbiota-derived signals is an important area for future investigation.

### The gut microbiota provides PRR ligands that can lead to IFN induction

Just like viruses, components of bacterial cells contain molecular patterns that can activate PRR signaling. Five different types of PRR can sense bacterial patterns, including TLRs, RLRs, NOD-like receptors (NLRs), DNA sensors, and AIM2-like receptors (ALRs). These bacterial PRRs are involved mainly in the induction of antibacterial signaling pathways, but a subset of these PRRs also induce IFN production during bacterial infection [40,41].

A recent study showed that TLR4 sensing of the outer membrane of *Bacteroides fragilis*, specifically the polysaccharide A (PSA) domain, leads to the induction of IFN-β by DCs in the colon lamina propria [9]. *In vitro* incubation of bone-marrow-derived DCs with PSA prior to vesicular stomatitis virus (VSV) infection results in a reduced percentage of infected DCs and increased cell viability. Oral administration of PSA to antibiotic-treated mice protects mice against infection with VSV or influenza A virus, increasing their survival in comparison to untreated controls [9]. This ability of the PSA of *B. fragilis* to induce protective TLR4-dependent IFN-β activity is distinct from TLR4 activation by the canonical *Escherichia coli* LPS ligand, which often leads to the induction of (excessive) proinflammatory responses [42,43]. Another nontoxic TLR4 ligand is the biopolymer poly-γ-glutamic acid (ϒ-PGA), which is produced by *Bacillus* sp. Similar to PSA, ϒ-PGA induces IFN-β which can inhibit MNV entry and replication *in vitro*, while oral administration of ϒ-PGA to MNV-infected mice results in increased serum IFN-β and reduced MNV levels in **Peyer’s patches** and **mesenteric lymph nodes** [44]. Altogether, these studies highlight the potential of microbiota-derived PRR ligands to induce protective IFN antiviral responses.

Intracellular sensing machinery was previously considered irrelevant for extracellular commensal bacteria and thought to be reserved for detection of invasive pathogens. However, several *in vitro* studies suggest that intracellular PRRs are also able to sense the nucleic acid of commensal bacteria and thereby lead to the induction of IFN-β expression. TLR3, which detects double-stranded RNA, is discriminately activated by commensal lactic acid bacteria (LAB), but not by pathogenic bacteria. dsRNA is uncommon in bacteria and is synthesized only by specific species under certain conditions, a potential explanation behind the specific capacity of LAB to induce TLR3 activation [45]. In a separate study, LAB have been shown to induce IFN-β via cGAS-stimulator of interferon genes (STING) and RLR mitochondrial antiviral signaling protein (MAVS) activation in human macrophages. Both sensors recognize cytosolic DNA and RNA, respectively, and are classically associated with viral infections [46]. Intriguingly, the ability of individual strains of LAB to induce type I IFN is inversely correlated with their ability to induce NF-κB, suggesting strain specificity in inducing pro- or anti-inflammatory responses [47]. Further work is required to determine whether these findings hold in *in vivo* experimental systems and if induction of IFN by intracellular sensors can provide protection against viral infections.

The involvement of bacterial microbiota ligands in the induction of IFN-λ is currently understudied. Early evidence, derived from *in vitro* stimulation of mouse bone-marrow-derived DCs and human epithelial cell lines with a variety of bacterial TLR ligands, has shown that ligands of commensal bacteria can induce type III IFN expression, especially through TLR5 [48]. Thus, the possibility that the gut microbiota signals through TLRs to induce type III IFN merits further investigation.

### The gut microbiota produces metabolites that can induce IFN production

Alongside being the source of PRR ligands, the gut microbiota produces a broad repertoire of metabolites that can act as key mediators of microbiota–host interactions. These metabolites are either a product of cometabolism of a dietary compound between the host and the gut microbiota or synthesized *de novo* in bacterial cells [49]. The potential of microbial metabolites to regulate host immunity is well recognized and has been reviewed comprehensively elsewhere [50,51]. Here, we discuss two classes of metabolites, short-chain fatty acids (SCFAs) and bile acids (BAs), that have been shown to modulate viral replication through IFN responses. Another microbial metabolite, desaminotyrosine (DAT), is also capable of inducing IFN responses resulting in reduced disease pathology despite negligible alteration in viral titer [52]. We anticipate that future research will uncover more relevant classes of microbiota-derived metabolites that can act as IFN modulators.

#### SCFAs

SCFAs are the product of fermentation of nondigestible dietary fiber by the gut microbiota. The composition of both host diet and anaerobic bacteria in the gut determine the SCFA profile of an individual. Acetate, propionate, and butyrate are the best-studied examples of SCFAs, and their role in shaping host immunity and physiology has been extensively described [53,54]. The immunomodulatory properties of SCFAs are ascribed to their function as **histone deacetylase** (**HDAC**) inhibitors, G-protein-coupled receptor (GPCR) agonists, and autophagy regulators [55].

SCFAs can modulate IFN responses to viral infections. Microbiota-derived acetate shows antiviral activity against RSV by increasing IFN-β in a GPR43-dependent manner. Mice administered either an SCFA-producer (species of the family Lachnospiraceae) or exogenous acetate exhibit reduced RSV pulmonary viral load, reduced migration of inflammatory cells into the lung, and overall improved survival [17]. Butyrate and propionate have been shown to have similar protective effects [17]. While the link between GPR43 engagement and IFN-β production needs further clarification, NF-κB activation has been implicated as evidenced by increased NF-κB p65 translocation to the nucleus following acetate supplementation [17].

Contrary to the capacity of acetate to restrict RSV replication in the mouse model, an *in vitro* study showed that butyrate supports the increased replication of several viruses [influenza A virus (IAV), reovirus, HIV, human metapneumovirus (hMPV), VSV] by reprogramming the expression of specific ISGs. The ability of butyrate to suppress ISG induction is suspected to be secondary to the HDAC-inhibitor properties of butyrate since treatment with a synthetic HDAC inhibitor also suppresses ISG expression, and a **histone acetyltransferase** (**HAT**) inhibitor reverses this suppression [16]. These contrasting proviral and antiviral effects may arise from different types of SCFA and virus tested. The use of immortalized cell lines may also drive divergence in research findings as transformed cells may have altered signaling pathways.

#### Bile acids

Primary BAs, such as cholic acid (CA) and chenodeoxycholic acid (CDCA), are synthesized by the host in the liver. Primary BAs undergo conjugation with either glycine or taurine to become water-soluble before being excreted to the small intestine. In the small intestine, the gut microbiota remove these amino acid groups and transform primary BAs to secondary BAs such as lithocholic acid (LCA) and deoxycholic acid (DCA) [36]. Therefore, commensal bacteria help to shape the composition of BAs in the gut through their biotransformative activity. In addition to having important functional roles in digestion and lipid absorption, BAs can signal through numerous metabolic pathways. The two best-described BA receptors are the Farnesoid-X-receptor (FXR), a nuclear receptor preferentially activated by primary BA, and Takeda-G-protein receptor 5 (TGR5), a membrane-bound receptor preferentially activated by secondary BAs [56,57].

Evidence describing BA modulation of IFN signaling pathways and subsequent viral infection is conflicting. Some studies have reported that BAs can negatively regulate induction of IFN signaling pathways, facilitating viral replication. For example, BAs are required components in calicivirus cell culture propagation systems owning to their ability to suppress STAT1 activation and thereby facilitate calicivirus replication [58]. BA treatment of hepatitis C virus (HCV) cell culture systems, using autonomously replicating HCV replicons in hepatoma cells, similarly reduces IFN-α/γ anti-viral activity and thereby increases HCV RNA and protein expression [59]. In contrast, BAs have been reported to block chikungunya virus (CHIKV) and MNV replication *in vivo* using mouse models through the induction of type I and III IFN, respectively [13,14]. Colonization of antibiotic-treated or GF mice with the commensal bacterium *Clostridium scindens*, which produces DCA, results in suppression of CHIKV viremia in serum and blood leukocytes in a type I IFN-dependent manner. Oral administration of purified DCA is sufficient to recapitulate this protective effect of *C. scindens* colonization, highlighting the involvement of secondary BAs in inducing IFN responses [14]. Similarly, antibiotic-treated mice colonized with *C. scindens* or supplemented with DCA exhibit induction of IFN-λ signaling that protects against MNV infection in the proximal gut [13].

How the signals from BAs integrate into IFN signaling pathways is currently unclear. BA receptors FXR and TGR5 are the most obvious suspects given the involved signaling pathways. Infections by some viruses, such as LCMV, herpes simplex virus type 1 (HSV-1) and Sendai virus (Sev), can induce the expression of BA transporters, leading to BA accumulation in both hepatic and nonhepatic cell types [60–62]. This accumulation of BAs can subsequently induce type I IFNs through FXR and TGR5 activation. During LCMV infection in a murine model, CD8^+^ T cell-mediated destruction of LCMV-infected hepatocytes results in the release of BAs which then engage with FXR receptors in neighboring hepatocytes to induce the production of type I IFNs. In the absence of FXR receptors, type I IFN gene expression decreases, BAs accumulate, and immune cell migration is disturbed, all leading to a failure of LCMV control [62]. Similarly, intracellular BA accumulation following infection with HSV-1 and SeV in THP-1 cells activates the TGR5–β-arrestin–sarcoma (SRC) kinase pathway. Subsequently, SRC kinase phosphorylates important components of the IFN signaling pathways, including RIG-I, MAVS, STING, TBK1, and IRF3, and thereby induces the expression of IFN-β [61]. It has been suggested that TGR5 may itself be an ISG because its expression is increased upon viral infection or IFN-β stimulation in a STAT-1-dependent manner [60]. In contrast, a study using microbiota-derived BA showed that DCAs directly prime IFN-λ activation during stimulation with poly(I:C) or MNV infection, and that coincubation of DCAs and synthetic FXR agonists results in abrogation of DCA-dependent enhancement of IFN-λ [13]. TGR5 involvement was not tested in this system. More studies are needed to further clarify the roles of different BA receptors in the IFN response.

#### DAT

DAT is a product of commensal bacteria degradation of plant-derived polyphenol compounds (flavonoids) [63]. Administration of DAT into mice infected with influenza A virus protected mice from infection-associated mortality and morbidity but did not reduce viral titers [52]. DAT treatment reduced lung tissue immunopathology by augmenting the type I IFN response of phagocytic cells. Oral gavage with *Clostridium*, the commensal bacterium producing DAT, offered a similar protective effect [52]. Importantly, DAT treatment appears only to be protective when administered before infection occurs. DAT administration post-infection exacerbated disease outcomes instead [52].

## Perspective: translating gut microbiota–IFN–viral interactions to a human context

The past several decades of research have transformed the understanding and appreciation of the numerous roles that the gut microbiota can have in the host defense against viral pathogens. Divergent microbiota profiles associate with both resistance and susceptibility to viral infections [64]. Similarly, the composition of the gut microbiota is an important factor in modulating the immune response to viral vaccination [65,66]. Full understanding of how the human microbiota can protect hosts from viral disease is needed to harness the microbiota’s therapeutic potential – either through antiviral therapies or through improvements in viral vaccine responses. Currently, research linking the gut microbiota to viral infections in humans is sorely lacking, with a preponderance of correlative studies in which microbiota-dependent mediators of immune responses to protection from viral infection remain largely unknown. Mechanistic insights from *in vitro* and animal studies can help to guide the exploration of microbiota-dependent antiviral immunity in the human context. It is also important to note that the human gut microbiota is a complex environment with numerous kingdoms of microbes beyond bacteria which can also strongly regulate IFN responses (Box 2). In addition, other nonintestinal compartments also have their own microbiota communities that may modulate local and distal immune responses. The microbiota composition of the lower respiratory tract, for instance, is a better predictor of clinical outcomes in severe acute respiratory virus coronavirus 2 (SARS-CoV-2) infection than the composition of the gut microbiota [67,68]. Thus, in clinical settings, it is important to consider the contribution of both the intestinal and extraintestinal microbiota in shaping host immunity.

IFN responses have a long history as therapeutic targets for human viral disease. IFN-α/β has been used as an antiviral treatment for HCV and hepatitis B virus (HBV) infection, though drug resistance and drug toxicity are of concern [69,70]. IFN-λ has been compared to IFN-α/β in clinical trials as a potentially less toxic but therapeutically equivalent treatment for chronic HCV (NCT01001754^i^) and HBV infections (NCT01204762^ii^) [71,72]. In addition, IFN has been explored as a potential vaccine adjuvant. In preclinical testing, HSV-2 and HIV vaccines adjuvanted with IFN-λ confer improved protection against challenge with relevant viruses [73,74]. In light of the recent SARS-CoV-2 pandemic, there has been substantial interest in exploring both type I and III IFN as antiviral treatments (Box 3).

We propose that there may be several advantages to using microbiota-dependent induction of endogenous IFN responses in antiviral therapies and as vaccines adjuvants: (i) microbiota-induced IFN is localized; (ii) predefined microbiota stimulants may be able to augment IFN responses to specific virus infections; and (iii) microbiota-induced IFN expression may result in fewer side effects due to endogenous feedback loops controlling IFN production. However, before translation into human applications is possible, several basic science questions remain to be addressed.

The first key needed research area is the age-dependency of IFN responses. An example is with rotavirus infections. IFN responses to MRV infection are quite distinct between neonatal and adult mice: whereas neonatal mice require both IFN-λ and IFN-α/β to control infection, adult mice require IFN-λ only [75]. Considering that human infants and the elderly are the most susceptible to life-threatening respiratory and gastrointestinal virus infections, and that the microbiota changes dramatically with age [76–78], in-depth understanding of the maturation of IFN responses and the microbiota over the course of the human lifespan is crucial.

The second key needed research area is understanding the risk for autoimmunity. The gut microbiota may excessively prime IFN responses, resulting in unwanted T cell responses to harmless peripheral or self-antigens [10], manifesting as allergy or autoimmunity. The delicate balance between sufficient priming for robust antiviral response versus overstimulation resulting in autoimmune pathogenesis needs further study and fine-tuning.

The final key needed research area is a better understanding of microbiota interactions with IFN-λ. IFN-λ was discovered only in 2004, and studies relating to its fundamental biology and interactions with the microbiota have been limited. IFN-λ may be preferred as a human therapeutic over IFN-α/β owing to its ability to induce highly localized responses with less risk for proinflammatory excess. Major research gaps include whether induction of IFN-λ by PRR ligands from commensal bacteria is possible and sufficient to protect from viral infection. Further, the mechanism(s) by which microbiota-dependent IFN-λ is linked to adaptive immunity, especially in its potential role as a vaccine adjuvant, requires further investigation.

## Concluding remarks

**Figure 2.**
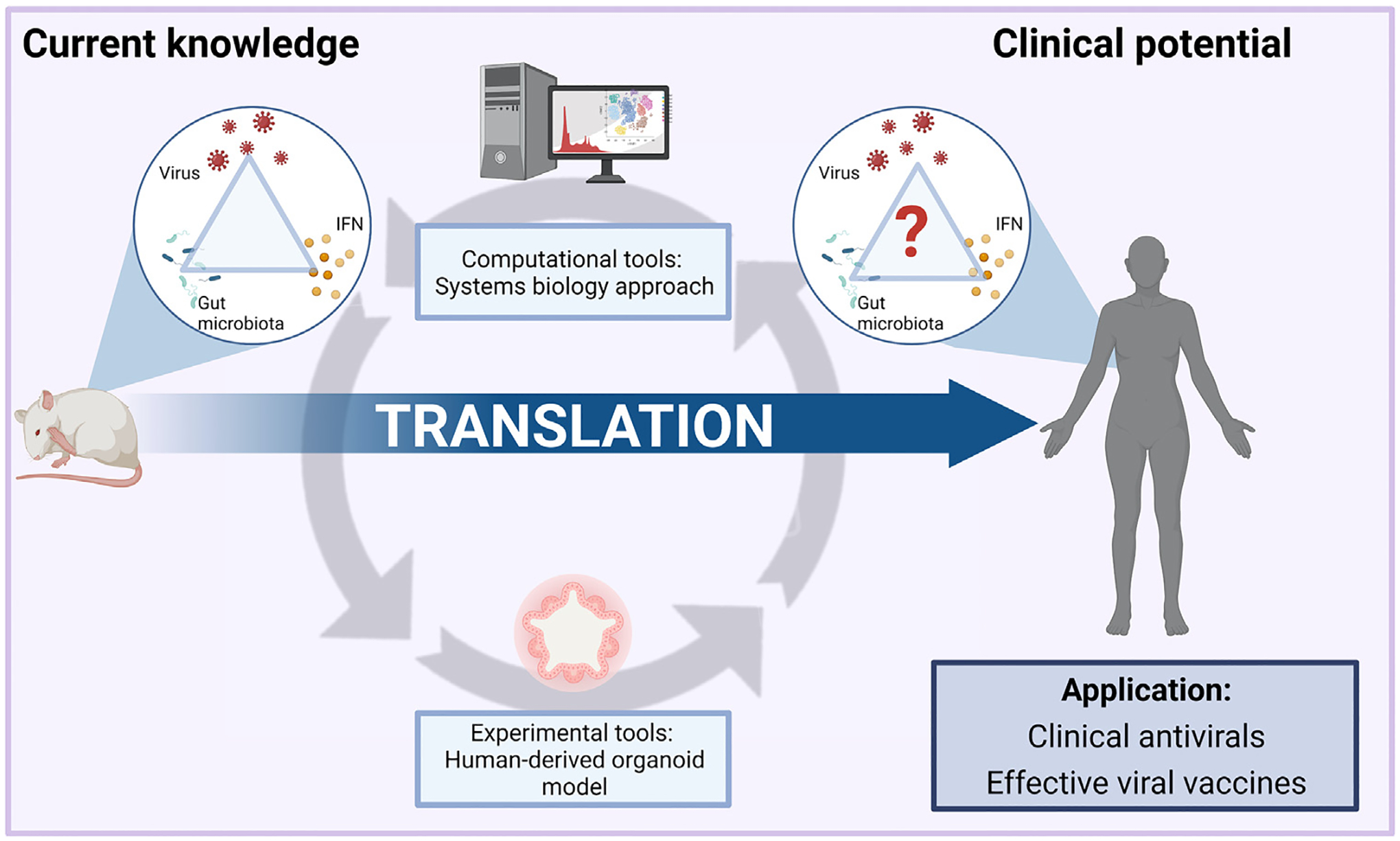
Future translation of microbiota–interferon(IFN)–viral interaction into the human context. The triangular relationship between the gut microbiota–host IFN response–viral infections has been extensively described in animal models. However, the translation of these interactions into the human setting for therapeutic purposes remains a major challenge. Recent computational tools, such as the systems biology approach and experimental tools such as human organoid platforms, may aid further exploration of this interaction, paving the way to microbiota-based therapies in viral control and viral vaccination. The figure was created with BioRender.com.

IFN modulation by the gut microbiota represents an exciting opportunity to harness microbiota-based therapeutic approaches for viral control. However, both basic and translational questions remain to be addressed before this vision can be realized (see Outstanding questions). The specific microbiota-derived signals that interact with IFN signaling pathways have been partially identified. Nonetheless, the possibility that other microbial components and metabolites, and even other kingdoms of the gut microbiota, may also induce a protective IFN response is deserving of further exploration. The regulation and function of microbiota-dependent IFN-λ in particular needs to be better understood. In addition, there is an urgent need for the field to move towards translation into the human setting. Recent advances in both computational approaches and experimental models (Box 4) have enabled more direct investigation of gut microbiota–host IFN–viral interaction in the human context, which can be leveraged in the near future for additional insights (Figure 2). The potential of microbiota-based therapy for viral control is promising, and IFN responses may serve as an important microbiota-regulated antiviral immunity component. The ability to regulate IFN responses in a specific and precise manner will allow the actualization of this promise.

## Figures and Tables

**Key figure. Figure 1.**
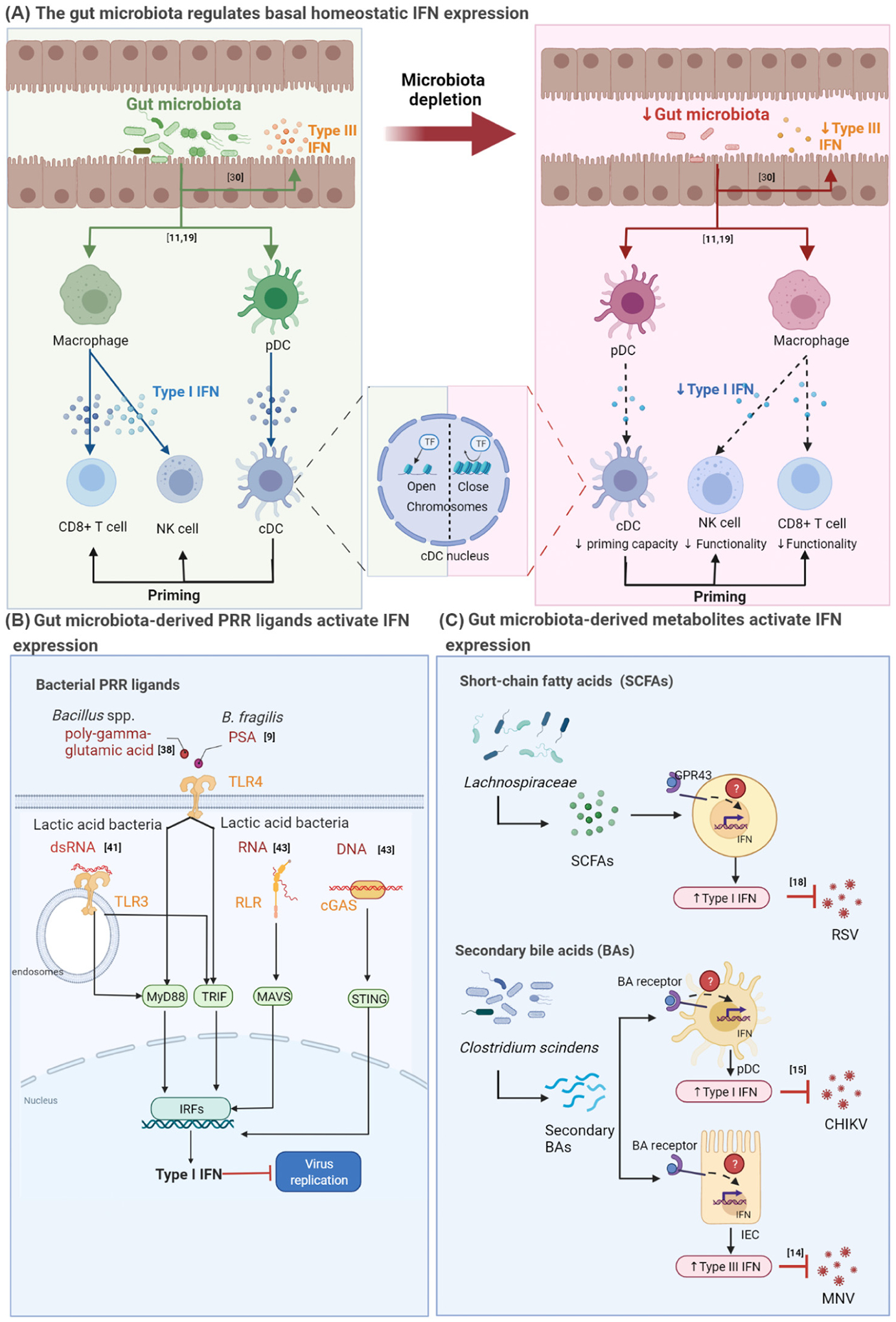
Potential mechanisms underlying microbiota regulation of interferon (IFN) antiviral immunity (A) The gut microbiota regulates basal homeostatic IFN expression. The gut microbiota can induce homeostatic type I IFN expression (shown in blue) from macrophages and plasmacytoid dendritic cells (pDCs) and homeostatic type III IFN (shown in orange) from intestinal epithelial cells (left panel). Type I IFN from macrophages is required for the priming of natural killer (NK) cell and CD8^+^ T cell function [18] (left panel). Type I IFN from pDCs is required for epigenetic programming of conventional dendritic cells (cDCs) so that they can prime NK cells and CD8^+^ T cells [19] (middle panel). When the gut microbiota is depleted, signals from the gut microbiota are diminished, leading to the reduction of basal homeostatic type I [18,19] and type III IFN, and impaired priming and functionality of cDCs, NK cells, and CD8^+^ T cells (right panel) [37]. (B) Gut microbiota-derived pathogen recognition receptor (PRR) ligands activate IFN expression. Components of the commensal gut microbiota generate molecular patterns that can bind to PRRs. For instance, *Bacillus* spp. poly-γ-glutamic acid [44] and *Bacteroides fragilis* polysaccharide A (PSA) [9] bind to TLR4, while the nucleic acids of lactic acid bacteria (LAB) can bind to either TLR3 [45], RIG-I-like receptors (RLRs) or cGAS [47]. This pattern recognition results in downstream signaling and type I IFN production. Depending on the type of PRR ligands and the PRR sensors, type I IFN production has been shown to block viral replication. (C) Gut microbiota-derived metabolites activate IFN expression. Gut commensals, such as members of the family Lachnospiraceae, can produce short-chain fatty acids (SCFAs) that can activate type I IFN expression in a GPR43-dependent manner to block the replication of respiratory syncytial virus (RSV) [17]. *Clostridium scindens* can transform primary bile acids (BAs) into secondary BAs. These secondary BAs can activate both the expression of type I IFN from pDCs to inhibit chikungunya virus (CHIKV) replication [14] and type III IFN from intestinal epithelial cells (IECs) to inhibit murine norovirus (MNV) replication [13]. cGAS, cyclic GMP-AMP synthase; IRFs, interferon regulatory factors; MAVS, mitochondrial antiviral-signaling protein; MYD88, myeloid differentiation primary response 88; TRIF, Toll–IL-1 receptor domain-containing adaptor inducing IFN-β; STING, stimulator of interferon genes. See references [9,11,15,18,19,30,38,41,43]. The figure was created with BioRender.com.

## Box 1. Examples of local and distal IFN antiviral activity modulated by the gut microbiota

The gut microbiota can modulate IFN-λ within the gastrointestinal tract during enteric viral infections [12,13,37]. One of the mechanisms is that the bacterial microbiota maintain the expression of a homeostatic tonic IFN-λ-mediated signature in intestinal epithelial cells (IECs). This is seen in murine rotavirus infections where depletion of the gut microbiota results in the loss of homeostatic tonic IFN-λ expression, limiting the host’s capacity to control murine rotavirus (MRV) infection [37]. In the proximal gut, commensal bacteria limit murine norovirus (MNV) infection, an inhibition associated with the production of microbiota-derived bile acids that prime IFN-λ [13]. Disruption of *Ifnlr1* (the receptor for IFN-λ) also limits MNV’s dependence upon bacteria, again implicating IFN-λ in antiviral regulation in the intestine [12]. Interestingly, the gut microbiota can also promote MNV infection in the distal intestine through an as-yet unclear mechanism [13]. A hypothesis is that the gut microbiota may express histo-blood group antigens (HBGAs) that are required for MNV infection [79]. These studies suggest that patterns of microbiota-induced IFN-λ expression may be location-dependent, but ultimately support a model in which the gut microbiota maintains or supports IFN-λ induction to limit enteric viral infection.

Gut microbiota modulation of type I IFN signaling at distal sites has been demonstrated in numerous murine studies in both germ-free (GF) and antibiotic-treated mice. A variety of extraintestinal virus infections have shown enhanced viral pathogenesis in the absence of microbiota-induced type I IFN. Reduced survival in antibiotic-treated mice upon infection with either LCMV [19] or VSV [9] is associated with reduced ISG expression in the spleen, a representation of the systemic immune compartment. During chikungunya virus (CHIKV) infection, viral loads in the spleen and serum of GF and antibiotic-treated mice are higher due to defective production of type I IFN by pDCs [14]. Similarly, absence of the gut microbiota leads to higher viral loads and lower type I IFN expression in the lung following infection of antibiotic-treated mice with influenza A virus [9,11] or respiratory syncytial virus (RSV) [17]. The influence of microbiota-induced type I IFN reaches as far as the brain, where reduced expression of IFN-β and ISGs in peripheral blood cells and spleen of antibiotic-treated mice leads to increased EMCV viral titers in the brain [15].

## Box 3. Lessons learned from the COVID-19 pandemic on IFN’s antiviral potential

SARS-CoV-2 and the COVID-19 pandemic have broadened our understanding of IFN’s potential as an antiviral therapeutic. Studies have had divergent findings. Some studies suggest that severe COVID-19 is associated with reduced and delayed IFN responses [90,91], while other studies implicate type I IFN, together with TNF and IL-1β, in hyperinflammation and progression to severe COVID-19 [92].

Clinical trials evaluating type I and III IFN therapies for patients hospitalized with COVID-19 have generally shown no or minimal clinical benefit [93–96]. The DISCOVERY trial (NCT04315948^iii^), the largest randomized controlled trial testing subcutaneous IFN-β1a in 2063 hospitalized COVID-19 patients, showed no mortality benefit compared to placebo [97]. However, in trials with smaller sample sizes, both type I (NCT04276688^iv^) [94] and type III IFNs (NCT04354259^v^) [95] appear to shorten the duration of viral shedding. To date, IFN therapy has not been broadly used in clinical COVID-19 practice.

Just as for other COVID-19 therapeutics, IFN’s clinical benefit may depend upon appropriate timing, route of administration, and use in appropriate patient populations [98]. Administering IFN early in disease was associated with a lower mortality in a retrospective cohort study [99]. A small randomized controlled trial of inhaled IFN treatment (NCT04385095^vi^) showed higher odds of improved clinical improvement than placebo [96]. Older adults infected with SARS-CoV-2 may benefit more from IFN therapy as they have a disturbed IFN expression pattern, consisting primarily of IFN-α, IFN-β, and IFN-λ2, in contrast to infected children, who predominantly express IFN-λ1 [100]. Type III IFN may also offer a more protective response compared to type I IFN. The IFN profile of patients with milder COVID-19 is dominated by expression of IFN-λ1 and IFN-λ3, while patients with more severe disease have higher type I IFN and IFN-λ2 [102]. Moreover, the antiviral state induced by type III IFN is confined to mucosal sites, potentially preventing excessive proinflammatory responses that may be induced by type I IFN [103].

Finally, as an alternative to exogenous IFN administration, fine-tuning endogenous IFN production by modulating the gut microbiota composition may hold potential. The human gut microbiome has the capacity to modulate the production of inflammatory cytokines [104], and enhanced inflammatory cytokines in severe COVID-19 patients has been suggested to be induced by systemic bacterial products [105]. Thus, modulating the gut microbiota composition to favor the production of protective IFN responses may be an alternative for the control of SARS-CoV-2 infection.

## Box 4. Tools for studying microbiota–host IFN–viral interactions in the human context

The emerging fields of **systems immunology** and **systems vaccinology** offer promising approaches to identify relevant microbiota targets that interact with and influence human antiviral and vaccine immunity. Via a systems vaccinology approach, IFN reponse was identified as an important early innate immune signature correlating with strong vaccine-induced antibody production for both the seasonal influenza vaccine [106] and the BNT162b2 mRNA SARS-CoV-2 vaccine [107]. However, microbiota modulation through antibiotic treatment appears to have only minimal effects on systemic IFN regulation following seasonal influenza virus vaccination [108]. Future studies are warranted to investigate whether more subtle microbiota modulation impacts IFN responses for other vaccines. It is possible that the gut microbiota may be more tightly interlinked with enteric and mucosal vaccine immunity, and thus IFN responses at mucosal sites following viral vaccination warrant further investigation.

The emergence of **human organoids** as a model system for studying host–virus interactions has dramatically enhanced scientists’ capacity to study IFN responses in mucosal compartments in a human context. Both human intestinal and lung organoid systems have been deployed to study the antagonism between IFN responses and numerous viruses, including human norovirus [109], adenovirus [110], astrovirus [111], enterovirus 71 [112], human rotavirus [113], and SARS-CoV-2 [114]. Efforts are currently underway to increase the complexity of intestinal organoid models by integrating gut microbiota components, immune cells, and the enteric nervous system in order to better mimic the human intestinal environment [115,116]. These models provide a platform by which the triangular interactions of gut microbiota–host IFN response–viral infection can be investigated, permitting therapeutic translation to human contexts.

## Highlights

The gut microbiota modulates antiviral type I and III IFN responses, ultimately regulating viral infection outcomes.

The gut microbiota is capable of modulating the IFN antiviral response both in the gastrointestinal tract and at extraintestinal sites.

The gut microbiota controls homeostatic IFN tone in the infection-naïve state, preparing the host for timely activation of antiviral responses upon infection.

Both commensal bacteria-derived ligands and metabolites signal to different cell types to regulate IFN signaling pathways and thereby indirectly control viral infection.

The gut microbiota–IFN–virus axis holds therapeutic promise for antiviral therapies and viral vaccines in humans, but extrapolation to humans remains to be demonstrated.

## Outstanding questions

What is the role of the gut microbiota in regulating IFN-mediated immunity in humans? How does human microbiota variation contribute to differential responses to viral infection?

How do IFN responses develop along the trajectory of human life, and how does the gut microbiota contribute to this development?

What are the roles of other microbiota kingdoms, such as the virome, in the regulation of IFN antiviral immunity?

How can we delicately regulate endogenous expression of IFN for sufficient antiviral protection without overstimulation that can lead to autoimmune pathogenesis?

What are the benefits of endogenous stimulation by the gut microbiota as opposed to the use of exogenous IFNs for antiviral therapy and viral vaccine performance?

## Glossary

Epigenetic markers: alterations in gene activity and expression that do not involve alteration in the DNA sequence but rather the ‘packaging’ of the DNA. Epigenetic markers include, but are not limited to, DNA methylation and histone modification
Histone acetyltransferase (HAT): an enzyme involved in the acetylation of the histone tail, a mechanism for gene regulation. In general, acetylated histone is a marker of increased gene transcription
Histone deacetylase (HDAC): the opposite of HAT, this enzyme is involved in the deacetylation of the histone tail, a mechanism for gene regulation. In general, deacetylated histone is a marker of reduced gene expression
Human organoid model: a three-dimensional tissue culture system, derived from stem cells of donated human tissue or differentiated pluripotent stem cells, thereby closely recapitulating the *in vivo* physiology and function of the represented organ
IFN-stimulated genes (ISGs): an array of genes whose expression is induced upon activation of the IFN signaling pathway. Expression of ISGs leads to the activation of an antiviral state in infected and neighboring cells
Intestinal/gut microbiota: the trillions of microbes, composed of bacteria, viruses, archaea, fungi, protozoa, and helminths, that reside in the gastrointestinal tract of the host and serve important functions in regulating host physiology and metabolism
Lipopolysaccharide (LPS): the main component of the outer membrane of Gram-negative bacteria. LPS is considered a bacteria-associated molecular pattern and is often used as a proxy for bacterial infection
M cells: a type of epithelial cell, found in mucosal tissue, that has a specialized immune surveillance function
Mesenteric lymph node: a secondary lymphoid organ, located in the connective tissue that attaches the intestine to the abdominal wall (mesentery), which functions to drain (filter) lymphoid fluid from the intestinal tract
Pattern-recognition receptors (PRRs): receptors commonly found in sentinel cells such as macrophages and dendritic cells. The function of PRRs is to recognize molecular patterns of pathogens and initiate inflammatory signaling cascades as a response. Nonimmune cells are also equipped with PRRs to a lesser extent
Peyer’s patches: one of the lymphoid tissues in the gastrointestinal tract which consists of aggregated lymphoid follicles. Peyer’s patches are important for immune surveillance and induction of immune tolerance
Poly I:C (polyinosinic:polycytidylic acid): an analog of double-stranded RNA, often used as a synthetic ligand for numerous PRRs and as a proxy for viral infection
Systems immunology: an approach using mathematical and computational modeling to integrate multiple types of - omics data (such as metabolomics, transcriptomics, and metagenomics) to investigate the interconnected immune components and their interactions in a certain disease or environment
Systems vaccinology: a subset of system immunology where the integration of multiple omics data is used to unravel the complex molecular signatures of vaccine immunity
